# Northern Bobwhite (*Colinus virginianus*) Mitochondrial Population Genomics Reveals Structure, Divergence, and Evidence for Heteroplasmy

**DOI:** 10.1371/journal.pone.0144913

**Published:** 2015-12-29

**Authors:** Yvette A. Halley, David L. Oldeschulte, Eric K. Bhattarai, Joshua Hill, Richard P. Metz, Charles D. Johnson, Steven M. Presley, Rebekah E. Ruzicka, Dale Rollins, Markus J. Peterson, William J. Murphy, Christopher M. Seabury

**Affiliations:** 1 Department of Veterinary Pathobiology, College of Veterinary Medicine, Texas A&M University, College Station, Texas, United States of America; 2 Genomics and Bioinformatics Core, Texas A&M AgriLife Research, College Station, Texas, United States of America; 3 Department of Environmental Toxicology, Institute of Environmental and Human Health, Texas Tech University, Lubbock, Texas, United States of America; 4 Texas A&M AgriLife Extension Service, Dallas, Texas, United States of America; 5 Rolling Plains Quail Research Ranch, 1262 U.S. Highway 180 W., Rotan, Texas, United States of America; 6 Department of Biological Sciences, University of Texas at El Paso, El Paso, Texas, United States of America; 7 Department of Veterinary Integrative Biosciences, College of Veterinary Medicine, Texas A&M University, College Station, Texas, United States of America; BiK-F Biodiversity and Climate Research Center, GERMANY

## Abstract

Herein, we evaluated the concordance of population inferences and conclusions resulting from the analysis of short mitochondrial fragments (i.e., partial or complete D-Loop nucleotide sequences) versus complete mitogenome sequences for 53 bobwhites representing six ecoregions across TX and OK (USA). Median joining (MJ) haplotype networks demonstrated that analyses performed using small mitochondrial fragments were insufficient for estimating the true (i.e., complete) mitogenome haplotype structure, corresponding levels of divergence, and maternal population history of our samples. Notably, discordant demographic inferences were observed when mismatch distributions of partial (i.e., partial D-Loop) versus complete mitogenome sequences were compared, with the reduction in mitochondrial genomic information content observed to encourage spurious inferences in our samples. A probabilistic approach to variant prediction for the complete bobwhite mitogenomes revealed 344 segregating sites corresponding to 347 total mutations, including 49 putative nonsynonymous single nucleotide variants (SNVs) distributed across 12 protein coding genes. Evidence of gross heteroplasmy was observed for 13 bobwhites, with 10 of the 13 heteroplasmies involving one moderate to high frequency SNV. Haplotype network and phylogenetic analyses for the complete bobwhite mitogenome sequences revealed two divergent maternal lineages (*d*_XY_ = 0.00731; *F*_ST_ = 0.849; *P* < 0.05), thereby supporting the potential for two putative subspecies. However, the diverged lineage (n = 103 variants) almost exclusively involved bobwhites geographically classified as *Colinus virginianus texanus*, which is discordant with the expectations of previous geographic subspecies designations. Tests of adaptive evolution for functional divergence (MKT), frequency distribution tests (*D*, *F*_S_) and phylogenetic analyses (RAxML) provide no evidence for positive selection or hybridization with the sympatric scaled quail (*Callipepla squamata*) as being explanatory factors for the two bobwhite maternal lineages observed. Instead, our analyses support the supposition that two diverged maternal lineages have survived from pre-expansion to post-expansion population(s), with the segregation of some slightly deleterious nonsynonymous mutations.

## Introduction

The northern bobwhite (*Colinus virginianus*; hereafter bobwhite) is one of 32 species belonging to the family Odontophoridae (New World quail), with wild populations historically ranging throughout the United States of America (i.e., USA, U.S.), Mexico and parts of the Caribbean [1, 2]. To date, more than 19 bobwhite subspecies have been named based on variation in size (decreasing from north to south) and male plumage [1, 2], with females displaying more similar plumage regardless of putative subspecies classification or geographic distribution [1, 2]. Relevant to wild bobwhite populations in the southern U.S. and northern Mexico, four subspecies have been recognized west of the Mississippi River, which include the eastern (*C*. *v*. *virginianus*), plains (*C*. *v*. *taylori*), Texas (*C*. *v*. *texanus*), and masked bobwhite (*C*. *v*. *ridgwayi*) [2, 3]. Among these, the male masked bobwhite is unequivocally the most phenotypically and geographically distinct (i.e., black head; Sonora, Mexico); with the eastern, plains, and Texas bobwhites exhibiting more subtle variation in male plumage and body size [2, 3].

The natural abundance of wild bobwhites in the U.S. has historically been observed to follow a boom-bust pattern, often with substantial annual variation observed [4–7]. Previous studies utilizing either breeding bird surveys or Christmas bird count data reported bobwhite declines more than 20 years ago [8–13], which is a range-wide population trend in the U.S. that remains ongoing today [14, 15]. The precise reasons for these broad-scale declines are likely complex, and have previously been attributed to variation in annual rainfall [4–6], changes in land use or scale, along with the decline of suitable habitats [4, 7, 12, 13, 16], thermal tolerances of developing embryos during a period of global warming [17, 18], harvest intensity by humans [19–21], especially during drought conditions [6, 16], sensitivity to ecotoxins [22, 23], red imported fire ants (*Solenopsis invicta*) [24, 25], and most recently, parasitic eyeworms [26–28]. However, it should also be noted that the same species of parasitic eyeworm (*Oxyspirura petrowi*) detected in wild bobwhites has also been detected in songbird species (i.e., Northern Mockingbird, *Mimus polyglottos*; Curve-billed Thrasher, *Toxostoma curvirostre*) [29] which have not been reported to be experiencing similar broad-scale population declines (http://www.iucnredlist.org). The desire to mitigate U.S. bobwhite population declines has prompted both the translocation of wild bobwhites to fragmented regions of their historic range as well as attempts at restocking or population supplementation using pen-raised bobwhites. To date, neither of these approaches has been documented to be highly successful in regions where modern abundance is low [30–35]. At present, a need exists to examine the genetic relationships and overall levels of divergence within and between putative bobwhite subspecies as well as their extant U.S. populations; to enable informed management and restoration efforts. A recent bobwhite mitochondrial DNA (mtDNA) study for representatives of the four putative subspecies described west of the Mississippi River revealed a general lack of distinct phylogeographic structure, evidence for demographic expansion following the Pleistocene, and an apparent discordance between patterns of mtDNA diversity and subspecies designations; with all inferences based on the analysis of a 353 bp fragment of the bobwhite mitochondrial control region [3]. Given the availability of a draft nuclear and mitochondrial genome assembly for the bobwhite [36], large-scale genetic studies are both possible and warranted, especially considering the apparent decline of wild bobwhite populations across the majority of their historic U.S. range [8, 9, 12–15].

We generated complete mitochondrial genome (mitogenome) sequences for 51 bobwhites sampled from six discrete ecoregions across Texas and Oklahoma (USA), which included representative samples from two putative bobwhite subspecies (*C*. *v*. *texanus*; *C*. *v*. *taylori*). Thereafter, we evaluated whether small mitochondrial fragments (i.e., partial or complete D-loop) could accurately resolve and predict the true haplotype structure and relationships among our samples, as compared to using complete mitogenome sequences to perform the same analyses. We further tested this same hypothesis with respect to accurately inferring historical patterns of demography, signatures of population substructure, and whether or not partial or complete mitogenomic data would support the presence of two or more putative bobwhite subspecies. The results of this study provide new insights regarding the demographic history and diversity of bobwhite maternal lineages west of the Mississippi River, but also clearly underscore the need for large-scale genomic studies in declining wildlife species.

## Results and Discussion

### Bobwhite Mitogenome Sequencing, Reference Mapping, and Variant Detection

Herein, we generated complete mitogenome sequences for 51 bobwhites representing two U.S. states (TX, OK) and 6 discrete ecoregions using standard Illumina paired-end (PE) sequencing technologies (i.e., TruSeq PE 2 x 100 bp; Illumina HiSeq2500; see Methods). Thereafter, we used these sequences and one additional bobwhite mitogenome (GenBank KJ914548.1) obtained from a phylogenetic study of the Odontophoridae (New World quail) [37] to predict single nucleotide variants (SNVs) and insertion-deletion mutations (indels) via reference mapping and alignment to an updated bobwhite mitogenome reference sequence [36] (n = 53 total bobwhite mitogenomes; see Methods). Using a probabilistic variant detection algorithm previously described [36] (see Methods), we predicted 344 segregating sites corresponding to 347 total mutations (n = 338 SNVs, 8 indels, and 1 multi-nucleotide variant, MNV), which included 49 putative nonsynonymous SNVs distributed across 12 protein coding genes. The majority of the nonsynonymous SNVs (i.e., 80%) were predicted at relatively low frequencies (i.e., < 0.10), and *ND4L* was the only mitochondrial protein coding gene for which no nonsynonymous variation was predicted. However, eight of the nonsynonymous SNVs were predicted at moderate frequencies (i.e., > 0.10) in our samples, with corresponding amino acid replacements predicted in five other mitochondrial protein coding genes (i.e., *CYTB*, *COX1*, *ATP6*, *COX3*, *ND5*). Summary data for all bobwhite mitogenome variants, including their genomic positions, average coverage, frequency distribution, average quality scores, and putative functional consequences (i.e., predicted amino acid replacements) are described in S1 Table. Similar to several previous avian and reptile studies, we also found an unambiguous *ND3* single nucleotide insertion (i.e., frameshift) in all bobwhite mitogenome sequences that were generated during this study (for review see [37–45]). Moreover, we also compared all predicted variants and their proximal flanking sequences to the known galliform nuclear mitochondrial sequences (numts) previously described [46], which included those identified in the first-generation draft genome assembly for the bobwhite [36]. With the exception of *ND3* [37–45], no indels or premature stop codons were observed in any bobwhite mitochondrial protein coding genes. However, two discrete SNVs were observed which could not be unequivocally excluded as potential numts (S1 Table), and therefore, we excluded these from all subsequent analyses.

### Bobwhite Mitogenome Heteroplasmy

The ability to generate bobwhite mitogenomes with deep coverage using Illumina PE sequencing technologies provided an opportunity to investigate the potential for heteroplasmy [47, 48] (see S1 Table), which has been reported in several avian species [44, 49–54], with one study indicating that paternal leakage may be a key factor in the emergence of some avian heteroplasmies [50]. Microheteroplasmy, which is defined by rare (i.e., independent) mutations found among 1–2% of all intra-individual mitogenomes, is common among adult humans, and has led some researchers to postulate whether this mutational burden may be linked to aging as well as age-related diseases [55–58]. However, microheteroplasmy can be differentiated from gross heteroplasmy by the presence of moderate to high frequency (i.e., common) mutations observed among the mitogenomes recovered from a single individual and/or a discrete tissue [55–58]. We detected evidence for gross mitochondrial heteroplasmy in 13 of the 51 surveyed bobwhites (i.e., 25%), which is similar to the heteroplasmy rates (i.e., 24%) reported for a survey of five human populations [47], and those reported for the Crested ibis (*Nipponia nippon*) (i.e., 22%) [54]. Specifically, in 13 of the 51 surveyed bobwhites, we identified 16 moderate to high frequency heteroplasmies (i.e., heterozygous mitochondrial sites) with minor allele frequencies ranging from 22% to 46.6% (S1 Table). All 16 detected heteroplasmies involved single nucleotide variants (SNVs) possessing average quality scores > 32, with 14 of the 16 (87.5%) observed as singletons among our population samples. Ten of the 13 bobwhites were predicted to possess only one heteroplasmic SNV (i.e., two unambiguous mtDNA haplotypes), whereas the other three heteroplasmies involved two unambiguous (n = 3 bobwhites) intra-individual heteroplasmic SNVs. Two of the 16 detected heteroplasmic SNV sites (i.e., 2216 and 2418; S1 Table) were also individually observed as homozygous SNVs (i.e., on one or two different mtDNA haplotypes) in a second bobwhite sequenced during our population survey. The distribution of the 16 heteroplasmic sites included both coding and noncoding regions (i.e., tRNA-Val, D-Loop, *12S*, *COX2*, *ATP6*, *CYTB*, *ND1*, *COX1*, and *COX3*), with 8 SNVs that were predicted to encode amino acid substitutions (S1 Table). As previously described, four plausible biological mechanisms may facilitate heteroplasmy including: 1) Paternal leakage; 2) Maternal transmission/inheritance of heteroplasmic variants; 3) *De novo* mutations that occur during embryonic development; and 4) Somatic aging, with age-related accumulation of heteroplasmic variants [47, 48, 50, 55, 57, 58]. In the absence of bobwhite samples of known pedigree, we could not unequivocally attribute the observed heteroplasmies to either paternal leakage or maternal inheritance of heteroplasmic sites. However, an evaluation of all the bobwhite mtDNA haplotypes generated in this study provides sufficient information (i.e., via variable sites) to predict the expected signatures of DNA contamination (i.e., the expected heterozygous mtDNA sites resulting from mixed samples). No evidence of contamination was observed. We also examined the distribution of ages among all of the heteroplasmic bobwhites observed in this study, and found nearly equal proportions of both juveniles and adults, indicating that somatic aging is unlikely to explain the observed heteroplasmies. Additionally, heteroplasmy was detected for bobwhites representing both putative subspecies [1–3] (*C*. *v*. *taylori*, n = 7; *C*. *v*. *texanus*, n = 6; S1 Table) sampled from five ecoregions and two U.S. states (i.e., Southwestern Tablelands of TX and OK, Western Gulf Coastal Plain of TX, Central Great Plains of TX and OK, Southern Texas Plains of TX, High Plains of TX and OK). Summary data for all detected heteroplasmies are described in S1 Table. Notably, most instances of bobwhite heteroplasmy detected in this study (i.e., 10 / 13 = 76.9%) relates to the presence of two intra-individual mtDNA haplotypes that differ by one SNV, which most likely arose via paternal leakage, maternal transmission, or *de novo* mutation during embryonic development. Future studies that include larger sample sizes of known pedigree are needed to deduce the biological mechanism(s) underlying instances of gross heteroplasmy in the bobwhite.

### Bobwhite Population Structure, Phylogeography, and Historical Demography

**Fig 1:**
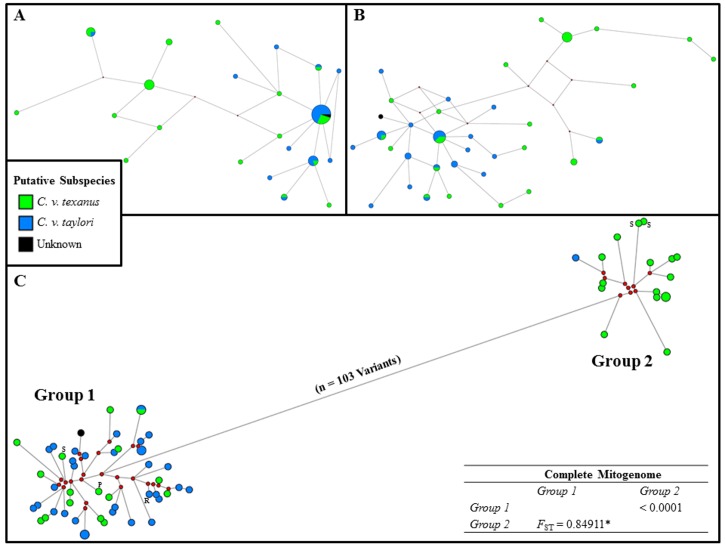
Median joining (MJ) haplotype networks [64] constructed for partial and complete bobwhite mitogenome sequences, with heteroplasmic minor allele haplotypes, and color-coded geographic subspecies designations [1–3]. (A) MJ haplotype network for 353 bp of the mitochondrial D-Loop [3] (n = 54, including 1 heteroplasmic minor allele haplotype). (B) MJ haplotype network for the complete D-Loop (1,152 bp; n = 55, including 2 heteroplasmic minor allele haplotypes). (C) MJ haplotype network for the complete mitogenome (16,709 bp including gaps; n = 66, including 13 heteroplasmic minor allele haplotypes). Default weights for SNPs and indels were used (10 and 20, respectively), with node sizes proportional to haplotype frequency, and branch lengths drawn to scale. Red dots indicate median vectors. The complete mitogenome haplotypes were observed to form two divergent clusters (i.e., Group 1, Group 2; n = 103 variants). Pairwise *F*_ST_ values (below diagonal) with standard errors (above diagonal) were computed to assess genetic differentiation between the two clusters, with the asterisk (*) indicating a significant *F*_ST_ value (*P* < 0.05). Fig 1C includes three complete mitogenome haplotypes for bobwhites lawfully harvested from active surrogating pastures (i.e., pen release sites = S), and one haplotype from a lawfully harvested pen-released bobwhite (P). (R) designates the reference mitogenome [36].

**Fig 2:**
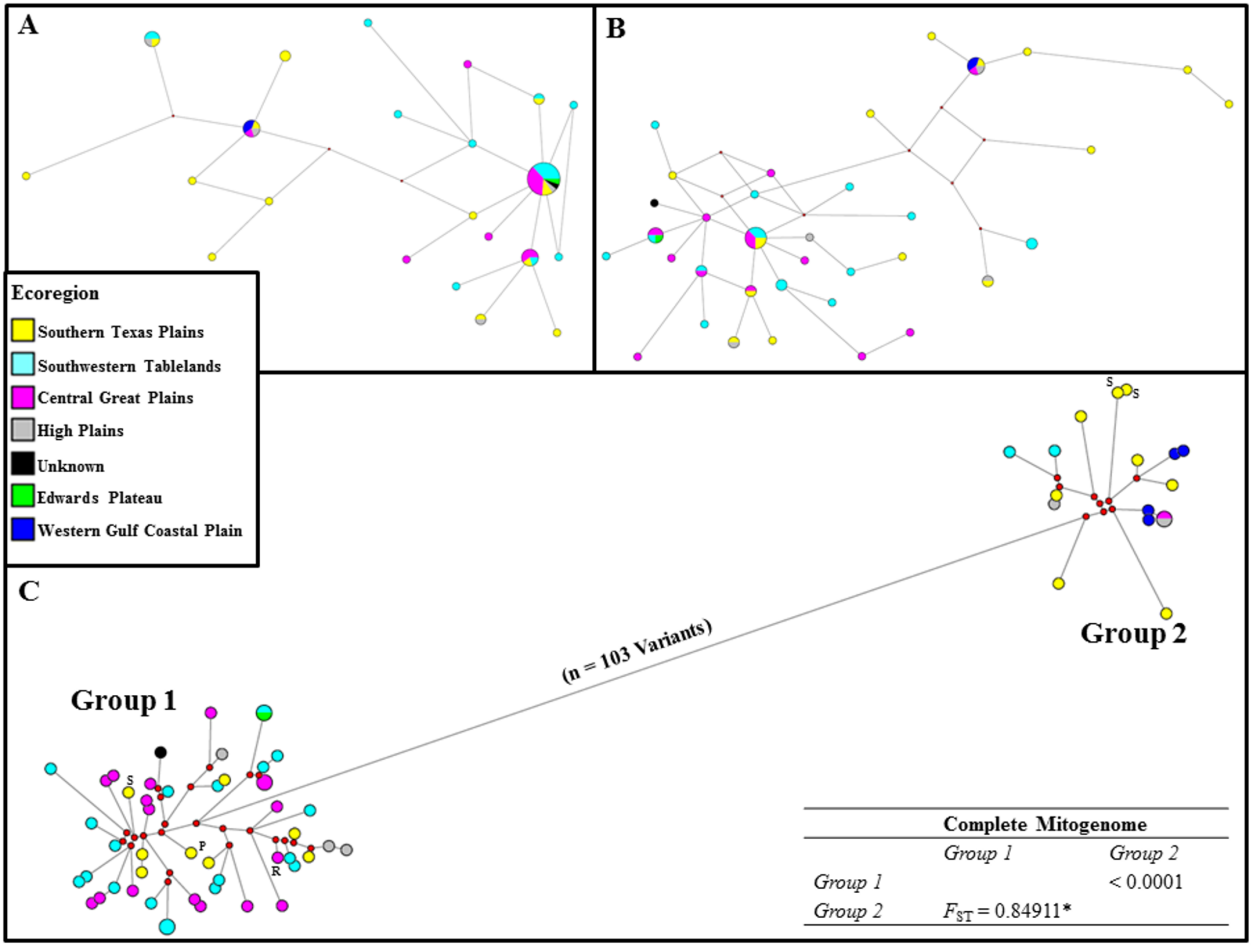
Median joining (MJ) haplotype networks [64] constructed for partial and complete bobwhite mitogenome sequences, with heteroplasmic minor allele haplotypes, and color-coded assignments to U.S. Environmental Protection Agency level III ecoregions (http://archive.epa.gov/wed/ecoregions/web/html/level_iii_iv-2.html). (A) MJ haplotype network for 353 bp of the mitochondrial D-Loop [3] (n = 54, including 1 heteroplasmic minor allele haplotype). (B) MJ haplotype network for the complete D-Loop (1,152 bp; n = 55, including 2 heteroplasmic minor allele haplotypes). (C) MJ haplotype network for the complete mitogenome (16,709 bp including gaps; n = 66, including 13 heteroplasmic minor allele haplotypes). Default weights for SNPs and indels were used (10 and 20, respectively), with node sizes proportional to haplotype frequency, and branch lengths drawn to scale. Red dots indicate median vectors. The complete mitogenome haplotypes were observed to form two divergent clusters (i.e., Group 1, Group 2; n = 103 variants). Pairwise *F*_ST_ values (below diagonal) with standard errors (above diagonal) were computed to assess genetic differentiation between the two clusters, with the asterisk (*) indicating a significant *F*_ST_ value (*P* < 0.05). Fig 2C includes three complete mitogenome haplotypes for bobwhites harvested from active surrogating pastures (i.e., pen release sites = S), and one haplotype from a lawfully harvested pen-released bobwhite (P). (R) designates the reference mitogenome [36].

Herein, we conducted a series of comparative analyses to determine whether similar population inferences or conclusions could be deduced from partial (i.e., partial or complete D-loop) and complete mitogenome nucleotide sequence data for 53 bobwhites (n = 6 ecoregions across TX and OK). As expected, haplotype diversity increases with the inclusion of increasing levels of mitogenomic sequence data, and nucleotide diversity decreases, the latter being due to the fact that nucleotide diversity is directly impacted by localized hyper-variability within short fragments of the mitochondria that are commonly targeted for population analyses (i.e., partial or complete D-Loop; See Table 1) (for review see [3, 59–63]). Median joining haplotype networks [64] constructed for partial bobwhite mitogenome sequences demonstrated an overt lack of resolution for drawing phylogenetic or population inferences in the bobwhite, as compared to networks constructed using complete mitogenome sequence data (Figs 1 and 2). Moreover, for analyses which utilized partial sequences, the reduction in mitochondrial genomic information content was observed to encourage spurious inferences in our samples (Table 1, Figs 1 and 2). For example, the total number of unique mitochondrial haplotypes and haplotype diversity were highly underestimated (i.e., collapsed) when partial mitogenome sequences were utilized, and therefore, some bobwhites wrongly appear to possess identical mitochondrial haplotypes (Table 1, Figs 1 and 2). This problem should be expected in many studies which utilize small mitogenome fragments, rather than complete mitogenome sequences. A comparative summary of all bobwhite mitochondrial analyses of diversity are presented in Table 1. Moreover, the true degree of mitogenome divergence and population structure among our sampled bobwhites was not detectable when popular mitogenome fragments (i.e., partial or complete D-Loop) (for review see [3, 59–63]) were analyzed (Figs 1 and 2). Nevertheless, similar to a previous bobwhite mitochondrial study [3], we did not observe strong phylogeographical clustering among the six surveyed U.S. Environmental Protection Agency (EPA) level III ecoregions (http://archive.epa.gov/wed/ecoregions/web/html/level_iii_iv-2.html; Fig 2). However, it should be noted that among the two discrete mitogenome haplotype groups detected (Figs 1 and 2), many of the diverged individuals (i.e., Group 2, Fig 2) originated from one ecoregion (n = 8 / 17, or 47%, Southern Texas Plains). The precise origin of this previously undetected diverged lineage [3], which represents approximately 25% of the total bobwhites surveyed in this study, is currently unknown.

**Table 1:** Bobwhite Mitochondrial Analyses of Diversity.

	Partial	Complete	Complete
Summary Data*	D-Loop	D-Loop	Mitogenome
Sample size (haplotypes)	54	55	66
Size of analyzed region (bp)	353 bp	1,152 bp	16,702 bp
Total variable sites	19	31	335
Total number of mutations	20	33	338
Total unique haplotypes	22	34	62
Haplotype diversity (Hd)	0.860	0.966	0.998
Nucleotide diversity (π)	0.00868	0.00435	0.00354

* Includes heteroplasmic minor allele haplotypes, excluding gaps.

**Fig 3:**
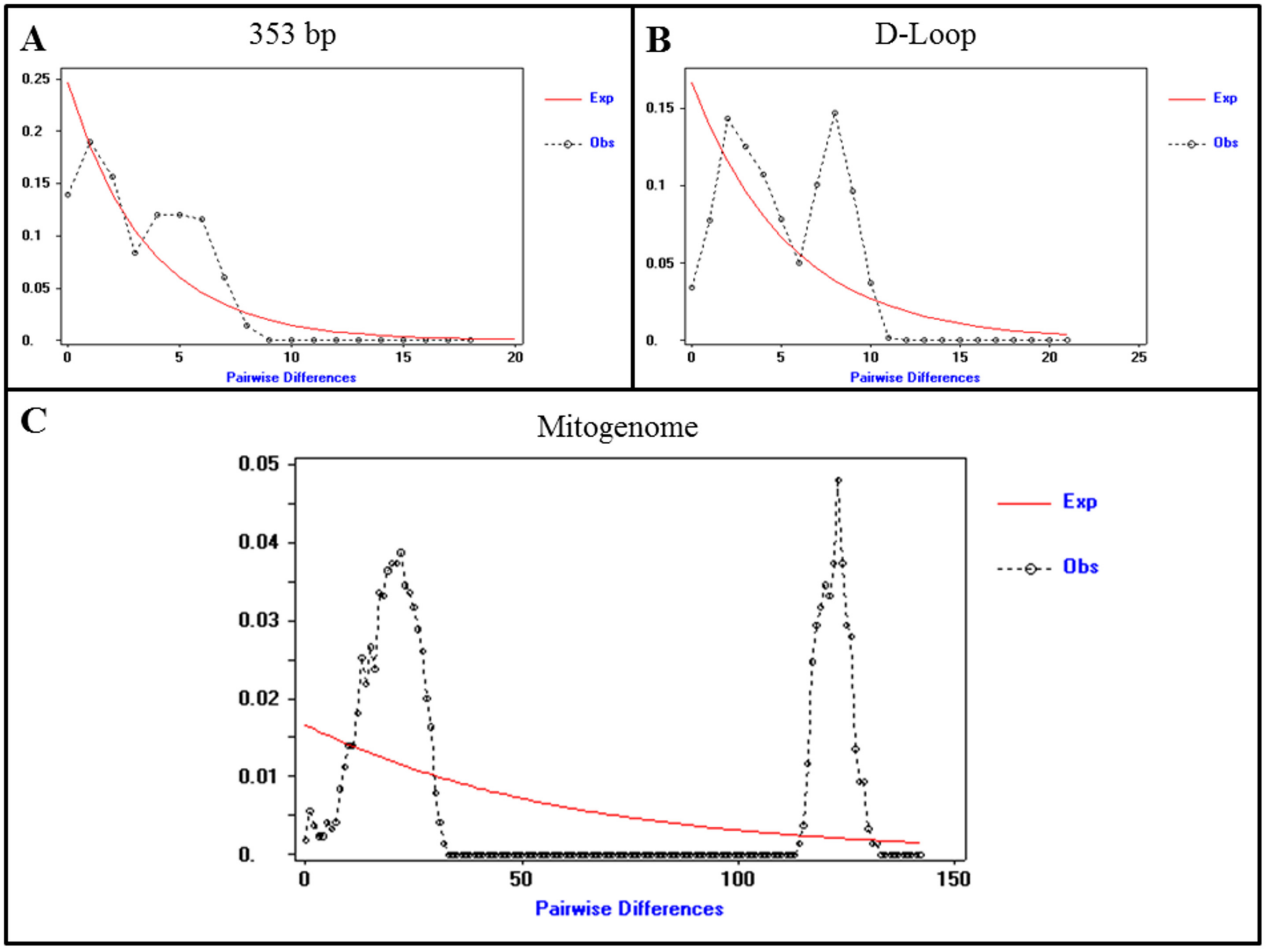
Bobwhite Mismatch Distributions. (A) 353 bp of the mitochondrial D-Loop [3] (n = 54, including 1 heteroplasmic minor allele haplotype). (B) Complete D-Loop (1,151 bp excluding gaps; n = 55, including 2 heteroplasmic minor allele haplotypes). (C) Complete mitogenome (16,698 bp excluding gaps; n = 66, including 13 heteroplasmic minor allele haplotypes). The x-axis represents the number of pairwise differences (mismatches) and the y-axis represents the frequency of these differences. The observed mismatch distribution (dashed line) is compared to the expected distribution (red line) for a stable population (i.e., constant population size).

Considering partial or complete bobwhite mitogenome sequences, we observed little support for the previously described geographic subspecies designations [1–3] across the six investigated ecoregions. However, complete mitogenome sequence analyses did reveal a modern bobwhite population structure that may potentially be comprised of at least two putative subspecies (*F*_ST_ = 0.849; *P* < 0.05; Figs 1 and 2); with the divergence between these two groups almost exclusively observed for a subset of bobwhites geographically classified as *C*. *v*. *texanus* [1–3] (Fig 1). Additional analyses further demonstrated statistically significant mitogenome differentiation and population subdivision between the two groups (i.e., *K*_S_, *K*_S_*, Z, Z*, *P* < 0.001 via permutation) [65]. The average number of nucleotide substitutions per site between the two lineages (Group 1 versus Group 2; Figs 1 and 2) was 0.00731 (*d*_XY_) [66], indicating that the average percent divergence was less than 1% (i.e., 0.7%). Collectively, 103 mitogenome mutations defined the split between the two bobwhite lineages within a median joining haplotype network (i.e., 101 SNVs, 2 Indels; Figs 1 and 2). Examination of all 103 network torso mutations revealed eight SNVs that were predicted to cause amino acid replacements, and 50% of these localized to *ND5* (S1 Table). No heteroplasmic variable sites were present in the network torso. Similar to our median joining haplotype networks, complete mitogenome divergence was also detected and visualized via mismatch distribution, where a bimodal distribution becomes overtly apparent with the inclusion of increasing levels of mitogenome sequence data (Fig 3). This bimodal distribution is in conflict with a previous bobwhite study that reported a unimodal mismatch distribution (i.e., based on a 353 bp mitogenome fragment), and corresponding inference suggesting a recent, rapid demographic expansion [3]. As shown in Fig 3, our analysis of the same 353 bp mitogenomic region produced a mismatch distribution that was strikingly similar to that of Williford and colleagues [3]. However, the true mismatch distribution corresponding to the extant maternal lineages sampled during this study was very poorly estimated when only 353 bp (i.e., partial D-Loop) were analyzed, indicating that more sequence data are necessary to correctly infer aspects of bobwhite historical demography and/or population substructure (Fig 3). Bimodal or multimodal mitochondrial mismatch distributions have not been uniformly interpreted in the literature; with some authors suggesting that these distributions reflect stable, stationary populations (i.e., post expansion) with or without spatial structuring [67–69], populations that are expanding spatially via few outward migrants per generation [70], or populations with tangible substructure and/or mutation rate heterogeneity (i.e., even while experiencing demographic expansions) [71, 72]. Therefore, the true biological origin(s) of any bimodal mismatch distribution may be complex. For example, factors such as biogeographical barriers [69], survival of some divergent lineages from a pre-expansion to a post-expansion population [73], the occurrence of historic population admixture [74], and even hybridization [75] have all been noted as likely origins. In this study, a comparison of all bobwhites geographically classified as either *C*. *v*. *texanus* or *C*. *v*. *taylori* [1–3] produced *F*_ST_ values that were statistically significant (*P* < 0.05; Table 2), which is concordant with a recent study [3], but notably, these *F*_ST_ values are far smaller than those obtained for a comparison of the two bobwhite mitogenome lineages elucidated by median joining haplotype networks (See Figs 1C and 2C; Group 1 vs Group 2). Although little evidence of strong phylogeographic clustering and corresponding subspecies distributions were observed in this study, and/or during a previous study [3], the *F*_ST_ values obtained via comparison of bobwhites that were taxonomically classified based on geographic subspecies designations [1–3] (Table 2; *C*. *v*. *texanus* versus *C*. *v*. *taylori*) suggests that perhaps a more pronounced substructure may have existed, historically, but has since been diluted. For example, the composition of extant wild bobwhite populations may potentially be affected by variation in the rates and patterns of dispersal, and/or an agricultural practice that involves the introduction of pen-reared lineages for restocking or supplementation [30, 33–35]. Herein we show that at least some pen-raised lineages are indistinguishable at the mitogenome sequence level from that of wild bobwhites (Figs 1C and 2C), while others have been distinguished using nuclear microsatellite loci [35]. Interestingly, annual survival and breeding of pen-reared bobwhites (pen-reared x pen-reared; pen-reared x wild) following release into suitable habitats has often been considered low [30, 33, 34]. However, some previous studies actually demonstrate either tangible annual survival rates [30, 35], and/or apparent reproduction (pen-reared x pen-reared; pen-reared x wild) [35]. Moreover, a study which previously concluded low post-release survival for pen-reared bobwhites, and subsequently discouraged their use for restocking, actually shows a basal survival rate that is greater than 20% across the entire observation period (i.e., 22 weeks) at one of the two study sites evaluated [30]. Therefore, it is apparent that some proportion of pen-reared bobwhites may successfully integrate into some wild populations [30, 35]. In this study, we produced complete mitogenome sequences for one hunter harvested pen-reared bobwhite (i.e., marked by a leg-band), and two bobwhites that were hunter harvested in a pasture routinely used for pen-reared releases. Complete mitogenome haplotypes corresponding to these three bobwhites were observed in both of the two divergent mitogenome clusters (n = 2 bobwhites in Group 1; n = 1 bobwhite in Group 2; Figs 1C and 2C), with the known pen-reared bobwhite possessing a haplotype that was more closely related to the most common wild bobwhite mitogenome sequences (i.e., Group 1; Figs 1C and 2C).

**Table 2:** Pairwise *F*_ST_ Values between Geographically Designated Bobwhite Subspecies.

	Partial D-Loop (353 bp)		Complete D-Loop (1,152 bp)		Mitogenome (16,709 bp)	
	*C*. *v*. *texanus*	*C*. *v*. *taylori*	*C*. *v*. *texanus*	*C*. *v*. *taylori*	*C*. *v*. *texanus*	*C*. *v*. *taylori*
*C*. *v*. *texanus*		≤ 0.0001		≤ 0.0001		≤0.0001
*C*. *v*. *taylori*	0.25407*		0.18956*		0.31271*	

* Significant (*P* < 0.05) *F*_ST_ values (below diagonal, with standard errors above diagonal).

**Fig 4:**
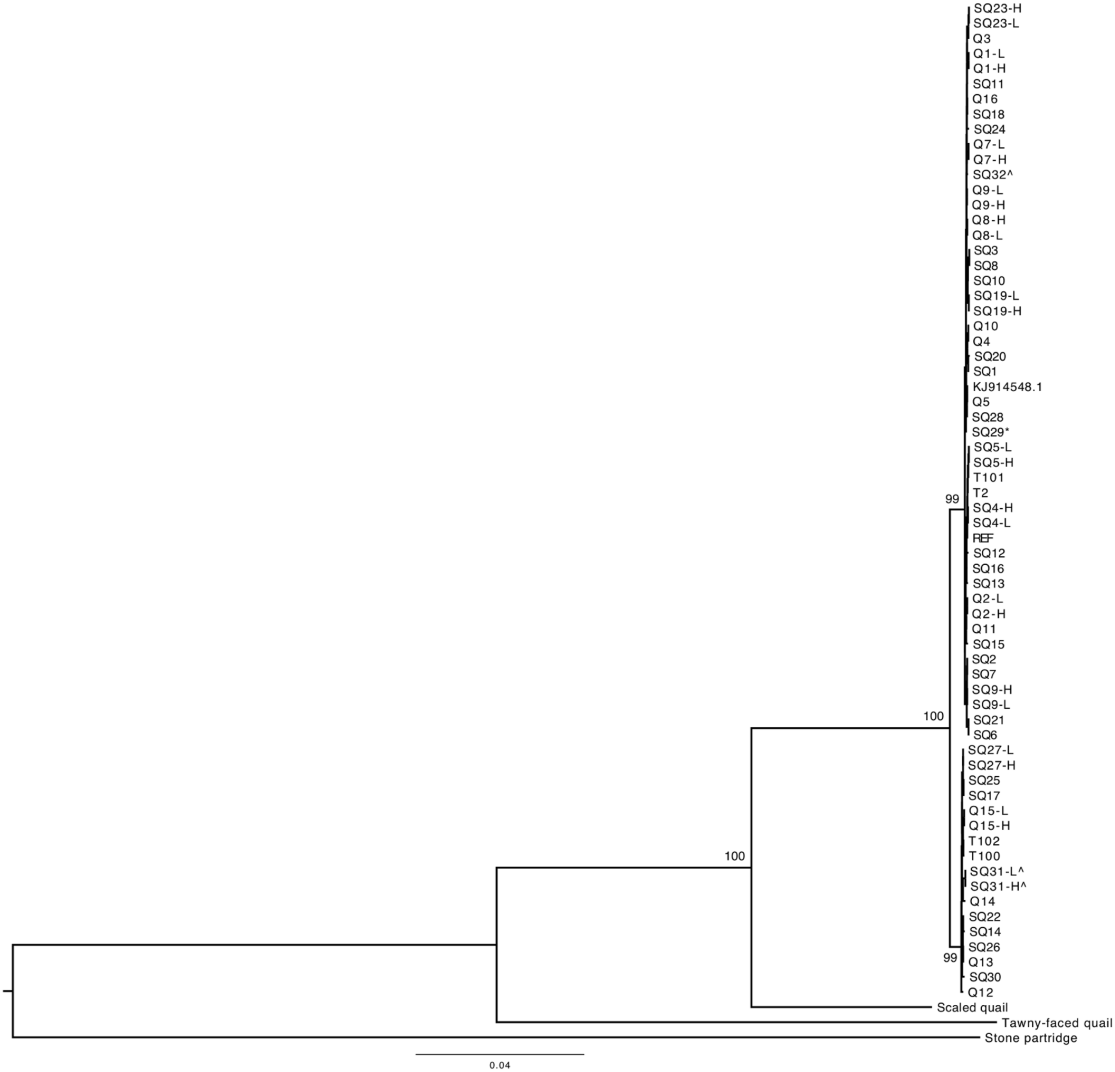
Maximum Likelihood-based Phylogeny Constructed with Expanded Taxon Sampling. Phylogeny of all bobwhite mitogenomes (n = 66, including 13 heteroplasmic minor allele haplotypes) in conjunction with mitogenomes for the scaled quail (*Callipepla squamata*), tawny-faced quail (*Rhynchortyx cinctus*) [37], and stone partridge (*Ptilopachus petrosus*) [37]. The asterisk (*) denotes a pen-released (n = 1) origin, and ‘^’ denotes bobwhites sampled from active surrogating pastures (i.e., pen release sites; n = 3). Terminal taxa noted with “H” and “L” refers to the high frequency (i.e., major) and low frequency (i.e., minor) heteroplasmic haplotypes, respectively. Individual bobwhites are labeled with laboratory identifiers (Q, SQ, T), with REF indicating the bobwhite reference sequence (GenBank Accession AWGT00000000.1), and KJ914548.1 indicating a bobwhite GenBank Accession [37] included in our analyses. The maximum likelihood phylogeny was constructed with RAxML 7.2.8 [96] using a GTR+Γ model of sequence evolution, with bootstrap support values based on 1,000 pseudoreplicates.

**Fig 5:**
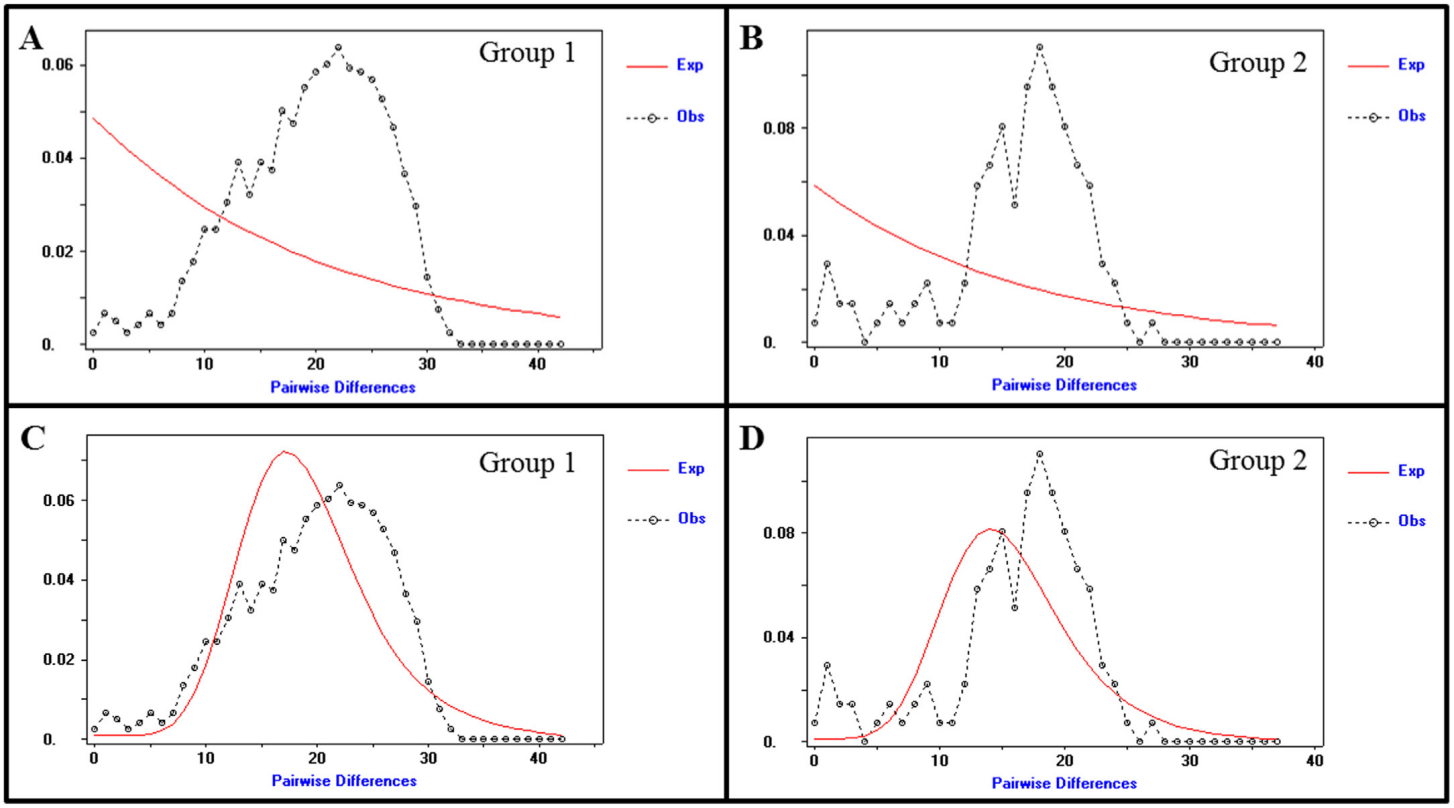
Bobwhite Historical Demography Inferred From Complete Mitogenome Sequences. (A) The observed mismatch distribution (dashed line) for bobwhite Group 1 (n = 49) as compared to the expected distribution (red line) for a stable population (i.e., constant population size). (B) The observed mismatch distribution (dashed line) for bobwhite Group 2 (n = 17) as compared to the expected distribution (red line) for a stable population (i.e., constant population size). (C) The observed mismatch distribution (dashed line) for bobwhite Group 1 (n = 49) as compared to the expected distribution (red line) for a growth-decline model. (D) The observed mismatch distribution (dashed line) for bobwhite Group 2 (n = 17) as compared to the expected distribution (red line) for a growth-decline model.

Since the majority of the diverged mitogenomes (i.e., Group 2, Figs 1C and 2C) were recovered from bobwhites that occupied overlapping ranges with the scaled quail (*Callipepla squamata*; also known as the blue quail), we investigated whether hybridization might be explanatory for the observed divergence. This hypothesis was predicated on previous observations that bobwhites and scaled quail may hybridize, both in the wild and in captivity [76, 77]. To address this question, we used standard Illumina PE sequencing technologies to produce a complete mitogenome sequence (n = 16,701 bp; GenBank Accession KT722338; see Methods) for a hunter harvested scaled quail that was obtained from the same ranch where multiple diverged bobwhites were sampled (Group 2, Figs 1C and 2C). Comparison of the scaled quail and bobwhite reference mitogenome revealed 1,215 mutational differences (n = 1,191 SNVs; n = 24 Indels), or greater than 7% divergence, indicating that hybridization between these two species is not explanatory for the two diverged bobwhite mitogenome haplotype groups (Figs 1C and 2C). Moreover, a maximum likelihood-based phylogeny constructed with expanded taxon sampling demonstrated that the scaled quail is more closely related to the bobwhite than the tawny-faced quail (*Rhynchortyx cinctus*) [78], and that both bobwhite haplotype groups were equidistant from the scaled quail (Fig 4). Likewise, using the scaled quail as an outgroup; Tajima’s relative rate test [79] revealed no significant rate heterogeneity between the two bobwhite lineages. These results are interesting because they suggest that neither bobwhite mitogenome group is more ancestral (or more derived) than the other, which supports the hypothesis that divergent maternal lineages have survived from a pre-expansion to a post-expansion population [73]. To further address this question, we examined the individual mismatch distributions for each bobwhite mitogenome group that was identified (i.e., Group 1, Group 2; Figs 1C and 2C). Both groups individually fit a demographic model of population growth-decline better than a model which assumed a stable, constant population size [80–82] (Fig 5). This result was robust to using either the mismatch distribution (i.e., pairwise number of differences) and/or the site frequency spectrum (i.e., segregating sites; not shown), and is generally concordant with previous reports of range-wide declines for the bobwhite (Fig 5) [8–14]. One final inference that could be drawn from our maximum likelihood-based phylogenies pertained to the likely origin of the observed bobwhite heteroplasmies. Specifically, in all instances of heteroplasmy, the two intra-individual mitogenome haplotypes were observed as sister taxa within the phylogenetic tree, thereby suggesting that either maternal transmission and/or developmental *de novo* mutation(s) were the most likely origin(s).

Application of Tajima’s and Fu’s test (i.e., *D*, *F*_S_) [83, 84] to each bobwhite mitogenome group (Figs 1C and 2C) revealed negative test statistics for both clusters (Group 1, n = 49, *D* = -1.83, *F*_S_ = -25.93; Group 2, n = 17, *D* = -1.25, *F*_S_ = -4.24). However, these tests were only statistically significant for Group 1 (i.e., via beta distribution and coalescent simulations; See Methods). Specifically, this result occurs due to an excess of rare variants and rare haplotypes in Group 1, which is consistent with new (i.e., young) mutations resulting from demographic expansion and/or natural selection (i.e., positive or purifying). Application of a multi-locus McDonald-Kreitman test [85, 86] (MKT, including all 13 mitochondrial protein coding genes and all haplotypes) to evaluate the possibility for functional divergence among the two bobwhite groups (Figs 1C and 2C) revealed no evidence for positive selection (*P <* 0.05; Overall mean proportion of adaptive substitutions (α) = -1.159). Notably, in this scenario, a significant multi-locus MKT results from the high proportion of polymorphism (including singleton heteroplasmic SNVs) as compared to the very low proportion of fixed (i.e., diverged) nucleotide sites between the two bobwhite mitogenome lineages detected (Figs 1C and 2C). This interpretation can be further evidenced by simply removing all heteroplasmic haplotypes possessing minor alleles (the majority of which were singletons), and again computing the multi-locus MKT, which revealed no evidence for selection (*P* > 0.05; (α) = -0.774). Likewise, standard MKT’s [85, 86] for all individual mitochondrial protein coding genes also revealed no evidence for positive selection and functional divergence (*P* > 0.05) regardless of the inclusion or exclusion of heteroplasmic mitochondrial haplotypes. Fine scale analyses conducted using Tajima’s test (*D*) via sliding window (i.e., 100 bp window, 25 bp step) also failed to produce any statistically significant evidence for natural selection within the mitochondrial protein coding genes for members of the two bobwhite mitogenome haplotype clusters (i.e., individual analyses for Group 1; Group 2). Similarly, application of Tajima’s and Fu’s Tests (*D*, *F*_S_) [83, 84] to the pooled set of all bobwhite mitogenome haplotypes (Group 1 + Group 2) also provided little support for strong selection, and less demographic insight relative to individual analyses carried out for members of each haplotype cluster (i.e., *D* = -0.59, *P* > 0.05; *F*_S_ = -16.24, *P* < 0.01 by coalescent simulations). Significantly negative values observed for *D* and *F*_S_ [83, 84] in relation to the Group 1 bobwhites (Figs 1C and 2C) most likely reflects signatures of demographic expansion in that lineage and/or purifying selection (i.e., perhaps detectable via larger Group 1 sample size). Application of Tajima’s test (*D*) to the pooled bobwhite sample (Group 1 + Group 2) using the sliding window method (i.e., 100 bp window, 25 bp step) revealed one mitogenome window located in the *ND5* gene (C-terminal region of NADH5; Pfam NADH5_C Domain) which departed from the neutral expectation (*D* = -1.83, *P* < 0.05). This result was driven by the occurrence of four singleton SNVs within the 100 bp window (n = 3 nonsynonymous; n = 1 synonymous). The distribution of these four singleton SNVs included haplotypes from both bobwhite mitogenome groups (Figs 1 and 2) identified by network analyses (n = 2 nonsynonymous and 1 synonymous in Group 1; n = 1 nonsynonymous in Group 2). Moreover, while the individual MKT [85, 86] for *ND5* of bobwhite Group 1 versus Group 2 (Figs 1 and 2) included four nonsynonymous substitutions that were diverged among the two mitogenome groups, that test was not statistically significant. Therefore, the collective results from our analyses of these data (i.e., pooled and by individual groups) are most concordant with the segregation of some slightly deleterious nonsynonymous mutations, as further evidenced by the negative overall mean proportion of adaptive substitutions (pooled), and the absence of statistical support for positive selection or functional divergence between the two bobwhite mitogenome lineages [85–88].

## Conclusions

For bobwhite samples included in the present study, utilization of small, popular mitochondrial fragments [3, 59–63] were observed to be largely insufficient to elucidate the true mitogenome haplotype structure and corresponding levels of divergence within a structured population. Likewise, discordant demographic inferences and failure to detect the extent of population substructure were also possible, as evidenced by small versus complete mitogenome sequence analyses. Our analyses of complete mitogenome sequence data for 53 bobwhites from six ecoregions across two U.S. states supported the potential for perhaps two putative subspecies that did not adhere to prior geographic designations [1–3], with the molecular caveat being that future nuclear genome analyses are also necessary to fully clarify bobwhite population structure and the genetic basis of all four putative subspecies west of the Mississippi River (USA). Collectively, our analyses of bobwhite mitogenomic data, including evidence for heteroplasmy, strongly support the deployment of low-cost, high-yielding, next-generation sequencing technologies in place of conventional PCR-based analyses of small mitochondrial fragments for future population studies. Additionally, population, demographic, and phylogenetic analyses reported in this study were robust to the inclusion or exclusion of heteroplasmic SNVs (Tables A-D and Figures A-F in S1 File). Finally, because next-generation sequencing technologies provide the opportunity to simultaneously capture both nuclear and mitochondrial DNA sequence information, the emergence and analysis of these types of data from relevant bobwhite populations are fully expected to compliment previous bobwhite microsatellite studies [89, 90].

## Methods

### Bobwhite and Scaled Quail Sampling, Taxonomy, and Isolation of Genomic DNA

Two sources of bobwhite quail (n = 25 females; n = 26 males) were utilized for DNA isolation in the present study, including lawfully harvested wild bobwhites for which ethical clearance is not applicable, and those collected via trapping, where ethical clearance is required. Bobwhites obtained via trapping (n = 27) were collected during two-week periods of August and October (2012 and 2013) using milo-baited funnel traps on private ranches and public wildlife management areas located in the Central Great Plains, Edwards Plateau, High Plains, and Southwestern Tablelands ecoregions of Texas and Oklahoma. Traps were designed to minimize injury and covered with natural vegetation in a manner that simulated natural loafing cover, which minimized stress by providing shade and concealment from predators, and also discouraged restless behavior. Following capture, and during processing, birds were kept in zippered pillow cases (i.e., darkened environment) to further ameliorate stress. A subset of all captured birds were euthanized by cervical dislocation, which is an American Veterinary Medical Association acceptable method for small birds. All birds were collected under authorization of a Texas Parks and Wildlife permit (SPR-1098-984; Austin, TX, USA) and via Institutional Animal Use Protocols from both Texas Tech University (IACUC 11049–07; Lubbock, TX, USA) and Texas A&M University (IACUC 2011–193; College Station, TX, USA). Skeletal muscle samples (i.e., from one or both legs) were obtained from bobwhites that were lawfully harvested (n = 24) on private ranches in the Central Great Plains, Southwestern Tablelands, Southern Texas Plains, and Western Gulf Coast Plain ecoregions of TX (USA). Likewise, skeletal muscle samples from the legs of one lawfully harvested scaled quail were also obtained from one of the same private ranches in the Southern Texas Plains ecoregion. Bobwhite ecoregion assignments followed the U.S. Environmental Protection Agency (EPA) level III ecoregion maps (http://archive.epa.gov/wed/ecoregions/web/html/level_iii_iv-2.html). Putative subspecies designations for all bobwhites included in this study (i.e., *C*. *v*. *texanus*; *C*. *v*. *taylori*) followed geographic designations described west of the Mississippi River (USA) [1–3]. Genomic DNA was isolated from skeletal muscle derived from the legs of bobwhites (n = 52) and one scaled quail (n = 1) using the MasterPure DNA Purification Kit, according to the manufacturer’s recommendations (Epicentre Biotechnologies Inc., Madison, WI) [36]. The presence of high molecular weight genomic DNA was assessed and confirmed by agarose gel electrophoresis, with quantitation via Nano Drop 1000 (NanoDrop Technologies Inc., Wilmington, DE), and by evaluating all isolates using a Qubit 2.0 fluorometer (Life Technologies Corp, Carlsbad, CA) [36].

### Illumina Library Construction and Sequencing

Small insert PE libraries were constructed using the TruSeq Nano LT Library Prep Kit (Illumina #FC-121-4001) according to the standard protocol supplied by the manufacturer. All quail PE libraries were multiplexed and processed using PE-125 cycle runs (2×125 bp), with data generation (i.e., image processing and base calling) occurring in real time on the Illumina HiSeq 2500v4 High Output instrument (Illumina Inc., San Diego, CA). Briefly, the sequencing strategy consisted of multiplexing four barcoded birds per lane, which delivered high mitochondrial coverage across all individual quail.

### Bobwhite Mitogenome Reference Mapping and Variant Detection

Prior to reference mapping, all Illumina sequence reads were trimmed for quality and adapter sequences using the CLC Genomics Workbench, as previously described [36]. Comparison of the initial bobwhite draft mitogenome reference sequence (GenBank Accession AWGT00000000.1) [36] with recently published mitogenome reference sequences for several members of the family Odontophoridae (New World quail) [37] revealed an in-frame gap in the *ND5* reference sequence; a complication related to *de novo* assembly of a circular mitochondrial chromosome into a linear contig (i.e., end gaps). However, the recent increase in mitogenome taxon sampling for species of the Odontophoridae [37] allowed for comparative correction and read-based validation of the gap within the previously reported bobwhite reference mitogenome sequence (GenBank Accession AWGT00000000.1) [36]. Thereafter, the trimmed Illumina sequence reads generated for 51 bobwhites were individually mapped to the corrected bobwhite mitogenome reference sequence [36] (i.e., equivalent in length to GenBank Accession KJ914548.1; mitogenome = 16, 702 bp) using the reference mapping algorithm within the CLC Genomics Workbench (v7.0.3 and v7.5.1) [36]. Reference mapping parameters were as follows: no masking; mismatch cost = 2; insertion cost = 3; deletion cost = 3; minimum read length fraction = 0.95; minimum fraction of nucleotide identity (similarity) = 0.95. Duplicate mapped reads were removed using the CLC Genomics duplicate mapped reads removal algorithm (version 1.0; For PCR-based libraries), which also aims to collapse reads that are only distinguished by minority-branch sequencing errors (http://www.clcbio.com/files/usermanuals/Mapped_Duplicate_Reads_Removal_Plugin.pdf). All mapped reads were extracted for deposition in the DRYAD digital repository (doi:10.5061/dryad.t39q6). Thereafter, we used the CLC probabilistic variant detection algorithm [36] to predict bobwhite mitogenome variants (SNVs, Indels, MNVs; http://www.clcbio.com/files/whitepapers/Variant_Caller_WP_web.pdf). This algorithm uses a Bayesian model and a Maximum Likelihood approach to calculate prior and error probabilities for the Bayesian model. These probabilities are used to determine the most likely allele combination per nucleotide position, with a user specified probability threshold for variant prediction equal to 0.95 [36]. Additional user specified settings for variant detection were as follows: ignore nonspecific matches = yes; ignore broken read pairs = no; minimum coverage = 4; variant probability ≥ 0.95; require variant in both forward and reverse reads = yes; maximum expected variants = 2; ignore quality scores = no. Resulting variant tracks for all 51 bobwhites were annotated by sequence overlap using mitogenome annotations previously described [36, 37], and the functional consequences of all putative variants were predicted using the vertebrate mitochondrial genetic code implemented in the CLC Genomics Workbench (v7.0.3 and v7.5.1). Consensus mitogenome sequences for all 51 bobwhites were manually constructed using individual variant reports, and then compared for accuracy to the consensus sequences computed by the CLC Genomics Workbench (v7.0.3 and v7.5.1).

### Generation of a Complete Scaled Quail Mitogenome Sequence

Two basic approaches were used to produce a complete scaled quail mitogenome. First, we mapped Illumina PE reads generated for a female scaled quail onto a bobwhite mitogenome reference sequence (GenBank Accessions AWGT00000000.1 and KJ914548.1) [36, 37] using the reference mapping algorithm implemented within the CLC Genomics Workbench (v7.5.1), and subsequently computed a scaled quail consensus. For comparison, all of the mapped reads were extracted and used in conjunction with the CLC *de novo* assembly algorithm, as previously described [36]. Both approaches produced identical results (16,701 bp), and the scaled quail mitogenome was comparatively annotated by sequence overlap(s) with mitogenome annotations previously described [36, 37]. Sequence overlaps were established by alignments performed in the CLC genomics workbench (v7.5.1) and/or via ClustalW online (http://www.ebi.ac.uk/Tools/msa/clustalw2/), with subsequent manual inspection. Translation of all 13 mitogenome protein coding genes provided no evidence for frameshifts, premature stop codons, or missing data (i.e., gaps).

### Population, Demographic, and Phylogenetic Analyses

For population analyses, all bobwhite mitogenome sequences were aligned using a multiple sequence alignment algorithm implemented within the CLC Genomics Workbench (v7.5.1; CLC alignment algorithm; Alignment available in Dryad doi:10.5061/dryad.t39q6). This alignment included one bobwhite mitogenome sequence of unknown geographic origin and/or putative subspecies designation (GenBank Accession KJ914548.1) [37] as well as the gap-corrected (*ND5*) reference mitogenome (GenBank Accession AWGT00000000.1) [36]. For bobwhites displaying unequivocal evidence of two intra-individual mitogenome haplotypes that differed by only one nucleotide (i.e., one heteroplasmic SNV; 10 / 13 bobwhites), both representative haplotype sequences were included in the alignment. Likewise, for bobwhites that displayed evidence for more than one heteroplasmic SNV (n = 3 bobwhites), we used allele frequency data to deduce the two putative intra-individual mitogenome haplotype sequences. Specifically, in all but one instance, the reference allele was observed at higher frequencies (i.e., by read count and coverage) than the alternative allele, thereby supporting the presence of one reference haplotype, and one alternative haplotype comprised of minor alleles (S1 Table). The only heteroplasmic SNV that deviated from this trend was excluded from further analyses. Likewise, two SNVs that could not be unambiguously excluded as potential numts were also removed from further analyses (S1 Table) [36]. Bobwhite median joining haplotype networks [64] were constructed using Network 4.6.1.3 (Fluxus Technology Ltd, Suffolk, England), with the default character weights for SNPs and indels (10 and 20, respectively). All networks were visualized and annotated within Network Publisher (Fluxus Technology Ltd, Suffolk, England), with manual adjustment of branch angles to ensure proper magnification and clarity without changing the branch lengths. Tests of population differentiation and subdivision (*K*_S_, *K*_S_*, Z, Z*) [65], estimates of haplotype and nucleotide diversity (Hd; π) [66], and bobwhite demographic models (i.e., constant stable population vs growth-decline) [80–82, 91] were computed in DnaSP version 5.10.01 [92]. Frequency distribution tests (*D*; *F*_S_) [83, 84] were also performed in DnaSP version 5.10.01 [92], with the significance of the test assessed by the beta distribution (*D*) and/or via coalescent simulation (*D*; *F*_S_) with 16,000 replicates. All computations performed in DnaSP version 5.10.01 [92] were based on the total number of mutations (excluding gaps), with one exception; the population growth-decline model was evaluated via mismatch distribution (i.e., pairwise number of differences) and the site frequency spectrum (i.e., segregating sites) [80–82, 91]. Pairwise fixation index (*F*_ST_) values were computed to assess genetic differentiation using a distance matrix in conjunction with a Tamura and Nei [93] model within the program ARLEQUIN v3.5.1.2 [94] (i.e., partial and complete D-loop; complete mitogenome). Both multi-locus and standard MKT’s were performed using the available web interface (http://mkt.uab.es/mkt/help_mkt.asp) with Jukes-Cantor correction [85, 86], with standard MKT’s [86] for individual mitochondrial protein coding genes also calculated in DnaSP version 5.10.01 [92]. Tajima’s relative rate test [79] was performed within the software program Mega v6.0 [95], and maximum likelihood phylogenies were constructed with RAxML 7.2.8 [96] using a GTR+Γ model of sequence evolution, with bootstrap support values based on 1,000 pseudoreplicates.

## Supporting Information

S1 File Comparative Analyses of Diversity for Partial and Complete Mitogenomes (Table A). Pairwise *F*_ST_ Values for Partial and Complete Mitogenomes of Bobwhites with Geographic Subspecies Designations (Table B). Diversity and Demographic Analyses for the Complete Mitogenomes of Bobwhites with Geographic Subspecies Designations, as Compared to Classification via Mitogenome Divergence (Table C). Genetic Differentiation among Divergent Bobwhite Mitogenome Lineages (Table D). Median joining (MJ) haplotype networks constructed for partial and complete bobwhite mitogenome sequences, excluding heteroplasmic minor allele haplotypes, and color-coded by geographic subspecies designations (Figure A). Median joining (MJ) haplotype networks constructed for partial and complete bobwhite mitogenome sequences, excluding heteroplasmic minor allele haplotypes, and color-coded by the U.S. Environmental Protection Agency level III ecoregions (Figure B). Mismatch distributions for partial and complete bobwhite mitogenome sequences, excluding heteroplasmic minor allele haplotypes (Figure C). Bobwhite historical demography, as inferred via mismatch distribution for constant population size and growth-decline models, excluding heteroplasmic minor allele haplotypes (Figure D). Texas bobwhite sampling locations with U.S. Environmental Protection Agency level III ecoregions (Figure E). Oklahoma bobwhite sampling locations with U.S. Environmental Protection Agency level III ecoregions (Figure F).
(PDF)

S1 Table Summary Data for all Bobwhite Mitogenome Variants. (XLSX)
