# Supplementary material for: Northern Bobwhite (Colinus virginianus) Mitochondrial Population Genomics Reveals Structure, Divergence, and Evidence for Heteroplasmy
Source: PLoS One. 2015 Dec 29;10(12):e0144913. doi: 10.1371/journal.pone.0144913 (PMC4699210; doi:10.1371/journal.pone.0144913)
Supplement: S1 File — Comparative Analyses of Diversity for Partial and Complete Mitogenomes (Table A). Pairwise F ST Values for Partial and Complete Mitogenomes of Bobwhites with Geographic Subspecies Designations (Table B). Diversity and Demographic Analyses for the Complete Mitogenomes of Bobwhites with Geographic Subspecies Designations, as Compared to Classification via Mitogenome Divergence (Table C). Genetic Differentiation among Divergent Bobwhite Mitogenome Lineages (Table D). Median joining (MJ) haplotype networks constructed for partial and complete bobwhite mitogenome sequences, excluding heteroplasmic minor allele haplotypes, and color-coded by geographic subspecies designations (Figure A). Median joining (MJ) haplotype networks constructed for partial and complete bobwhite mitogenome sequences, excluding heteroplasmic minor allele haplotypes, and color-coded by the U.S. Environmental Protection Agency level III ecoregions (Figure B). Mismatch distributions for partial and complete bobwhite mitogenome sequences, excluding heteroplasmic minor allele haplotypes (Figure C). Bobwhite historical demography, as inferred via mismatch distribution for constant population size and growth-decline models, excluding heteroplasmic minor allele haplotypes (Figure D). Texas bobwhite sampling locations with U.S. Environmental Protection Agency level III ecoregions (Figure E). Oklahoma bobwhite sampling locations with U.S. Environmental Protection Agency level III ecoregions (Figure F). (PDF) [file pone.0144913.s001.pdf]

Table A. Comparative Analyses of Diversity for Partial and Complete Mitogenomes.

**Summary Data***	**Partial D-Loop**	**Complete D-Loop**	**Complete Mitogenome**
Sample size (haplotypes)	53	53	53
Size of analyzed region (bp)	353	1,152	16,702
Total variable sites	19	31	323
Total number of mutations	20	33	326
Total unique haplotypes	21	32	49
Haplotype diversity (Hd)	0.855	0.963	0.997
Nucleotide diversity (π)	0.00858	0.00424	0.00358

* Excluding heteroplasmic minor allele haplotypes and gaps.

Table B. Pairwise F_{ST} Values for Partial and Complete Mitogenomes of Bobwhites with Geographic Subspecies Designations.

	**Partial D-Loop (353bp)**		**Complete D-Loop (1,152 bp)**		**Complete Mitogenome (16,709 bp)**	
	C. v. texanus	*C. v. taylori*	*C. v. texanus*	*C. v. taylori*	*C. v. texanus*	*C. v. taylori*
C. v. texanus		< 0.0001		≤ 0.0001		≤ 0.0001
C. v. taylori	0.24654*		0.19451*		0.29450*	

* Significant ($P < 0.05$) F_{ST} values (below diagonal, with standard errors above diagonal); excluding heteroplasmic minor allele haplotypes.

Table C. Diversity and Demographic Analyses for Complete Mitogenomes of Bobwhites with Geographic Subspecies Designations, as Compared to Classification via Mitogenome Divergence.

Subcategories *	**Hd**	$\boldsymbol{\pi}$	$\boldsymbol{D}$	$\boldsymbol{F_S}$
Group 1 + Group 2	0.997	0.00358	-0.6017	-8.845[c]
Group 1	0.996	0.00116	-1.8893[a,c]	-15.679[d]
Group 2	0.989	0.00097	-1.3933	-2.534
C. v. texanus	0.997	0.00430	0.3162	-2.258
C. v. taylori	0.994	0.00167	-2.2034[b,d]	-4.822

* Excluding heteroplasmic minor allele haplotypes.
[a] $P < 0.05$ (beta distribution)
[b] $P < 0.01$ (beta distribution)
[c] $P < 0.05$ (coalescent simulations)
[d] $P < 0.01$ (coalescent simulations)

Table D. Genetic Differentiation among Divergent Bobwhite Mitogenome Lineages.

	K_S	K_S*	Z	Z*
Value[‡]	18.55985	2.90158	397.34875	5.67334
P	< 0.001	< 0.001	< 0.001	< 0.001

[‡] Excluding heteroplasmic minor allele haplotypes.

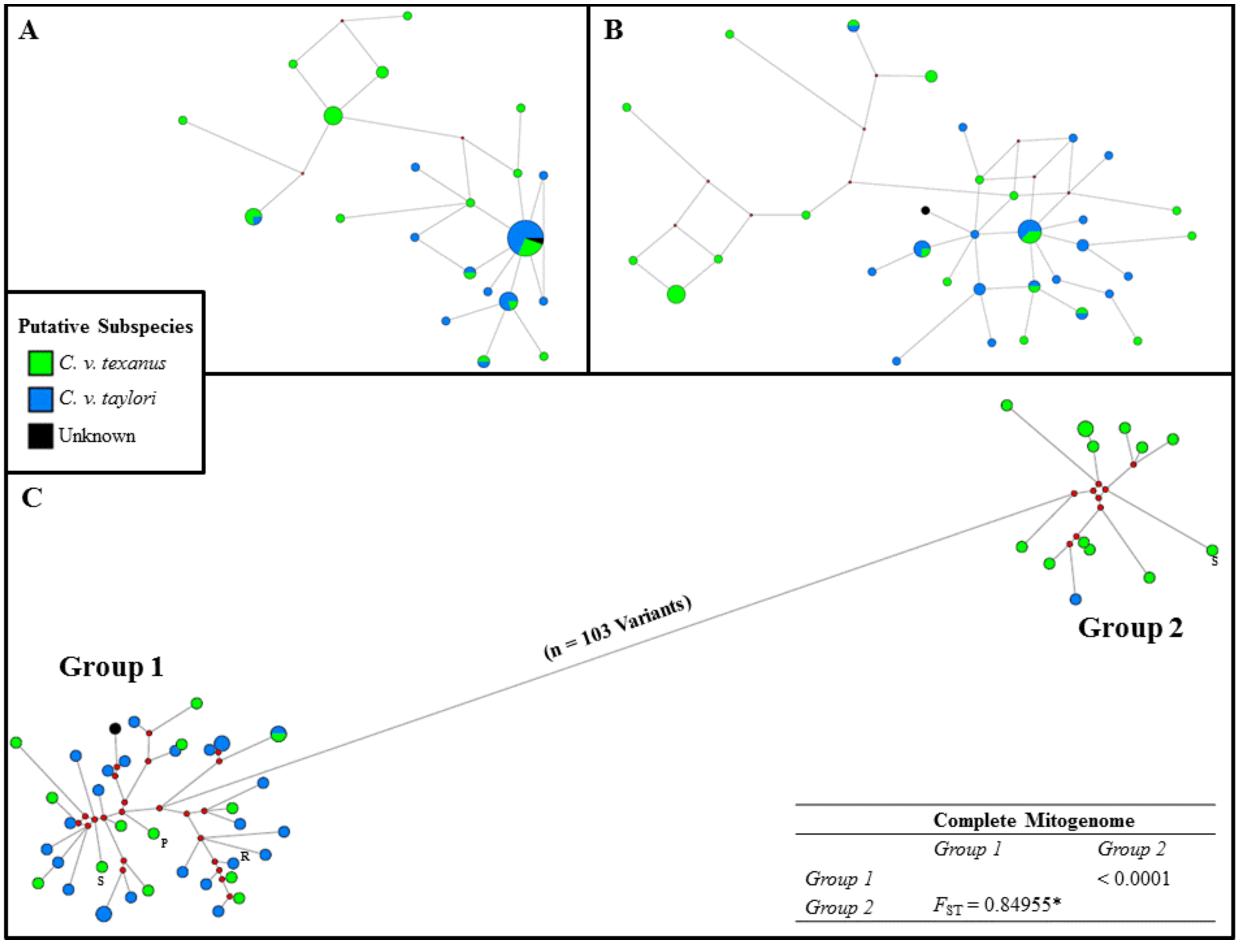

Figure A. Median joining (MJ) haplotype networks [64] constructed for partial and complete bobwhite mitogenome sequences, excluding heteroplasmic minor allele haplotypes, and color-coded by geographic subspecies designations [1-3]. (A) MJ haplotype network for 353 bp of the mitochondrial D-Loop [3]. (B) MJ haplotype network for the complete D-Loop (1,152 bp). (C) MJ haplotype network for the complete mitogenome (16,709 bp including gaps). Default weights for SNPs and indels were used (10 and 20, respectively), with node sizes proportional to haplotype frequency, and branch lengths drawn to scale. Red dots indicate median vectors. The complete mitogenome haplotypes were observed to form two divergent clusters (i.e., Group 1, Group 2; n = 103 variants). Pairwise F_{ST} values (below diagonal) with standard errors (above diagonal) were computed to assess genetic differentiation between the two clusters, with the asterisk (*) indicating a significant F_{ST} value ($P < 0.05$). Panel C includes two complete mitogenome haplotypes for bobwhites lawfully harvested from active surrogating pastures (i.e., pen release sites = S), and one haplotype from a lawfully harvested pen-released bobwhite (P). (R) Indicates the reference mitogenome [36].

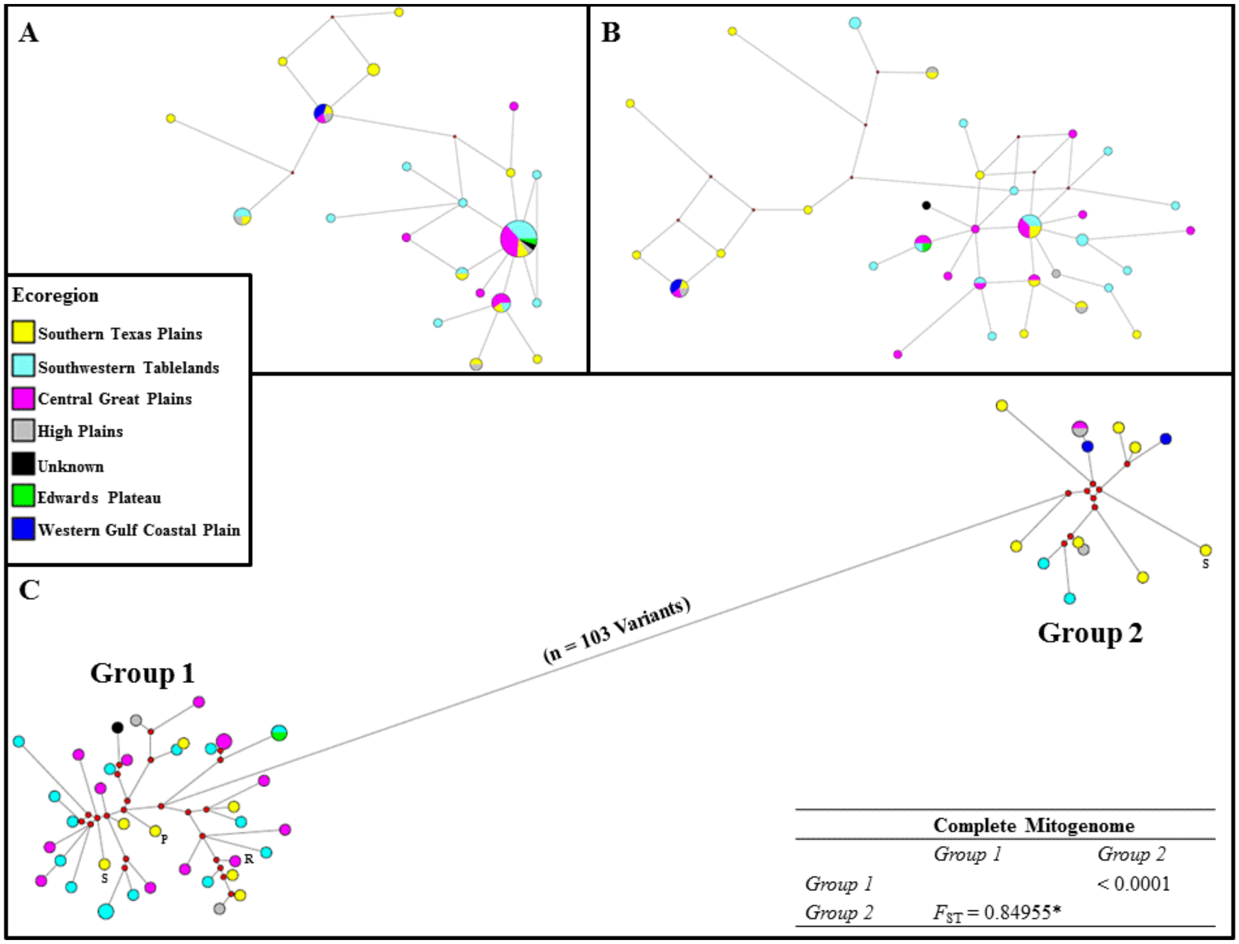

Figure B. Median joining (MJ) haplotype networks [64] constructed for partial and complete bobwhite mitogenome sequences, excluding heteroplasmic minor allele haplotypes, and color-coded by the U.S. Environmental Protection Agency level III ecoregions (http://archive.epa.gov/wed/ecoregions/web/html/level_iii_iv-2.html). (A) MJ haplotype network for 353 bp of the mitochondrial D-Loop [3]. (B) MJ haplotype network for the complete D-Loop (1,152 bp). (C) MJ haplotype network for the complete mitogenome (16,709 bp including gaps). Default weights for SNPs and indels were used (10 and 20, respectively), with node sizes proportional to haplotype frequency, and branch lengths drawn to scale. Red dots indicate median vectors. The complete mitogenome haplotypes were observed to form two divergent clusters (i.e., Group 1, Group 2; n = 103 variants). Pairwise F_{ST} values (below diagonal) with standard errors (above diagonal) were computed to assess genetic differentiation between the two clusters, with the asterisk (*) indicating a significant F_{ST} value ($P < 0.05$). Panel C includes two complete mitogenome haplotypes for bobwhites harvested from active surrogating pastures (i.e., pen release sites = S), and one haplotype from a lawfully harvested pen-released bobwhite (P). (R) Indicates the reference mitogenome [36].

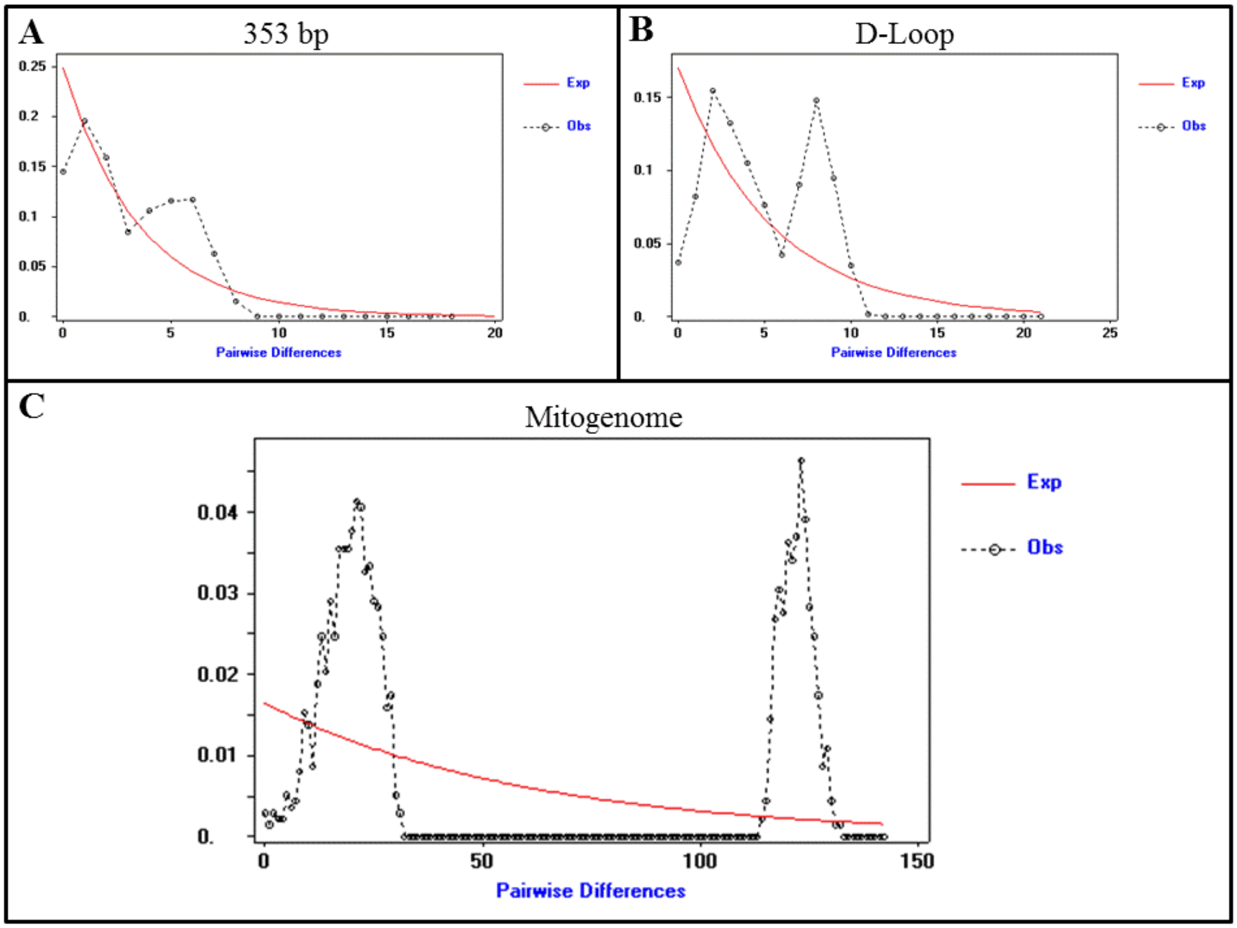

Figure C. Mismatch distributions for partial and complete bobwhite mitogenome sequences, excluding heteroplasmic minor allele haplotypes. (A) 353 bp of the mitochondrial D-Loop [3]. (B) Complete D-Loop (1,151 bp excluding gaps). (C) Complete mitogenome (16,698 bp excluding gaps). The x-axis represents the number of pairwise differences (mismatches) and the y-axis represents the frequency of these differences. The observed mismatch distribution (dashed line) is compared to the expected distribution (red line) for a stable population (i.e., constant population size).

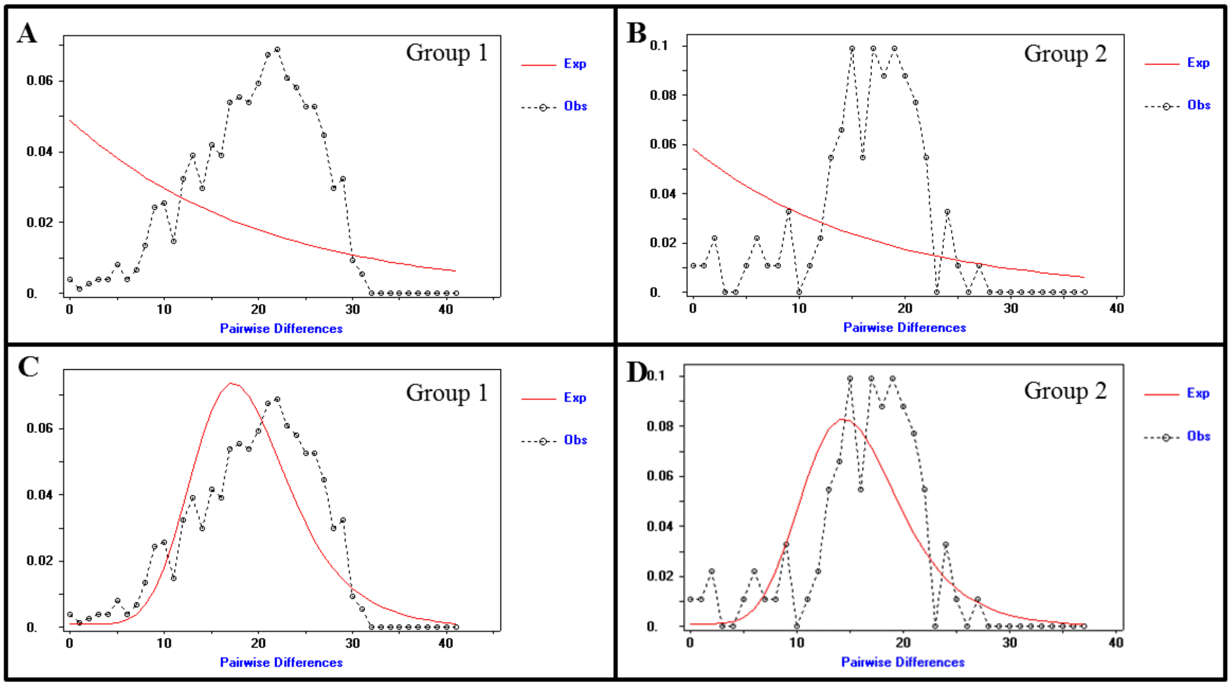

Figure D. Bobwhite historical demography, as inferred via mismatch distribution for constant population size and growth-decline models, excluding heteroplasmic minor allele haplotypes. (A) The observed mismatch distribution (dashed line) for bobwhite Group 1 (n = 39) as compared to the expected distribution (red line) for a stable population (i.e., constant population size). (B) The observed mismatch distribution (dashed line) for bobwhite Group 2 (n = 14) as compared to the expected distribution (red line) for a stable population (i.e., constant population size). (C) The observed mismatch distribution (dashed line) for bobwhite Group 1 (n = 39) as compared to the expected distribution (red line) for a growth-decline model. (D) The observed mismatch distribution (dashed line) for bobwhite Group 2 (n = 14) as compared to the expected distribution (red line) for a growth-decline model.

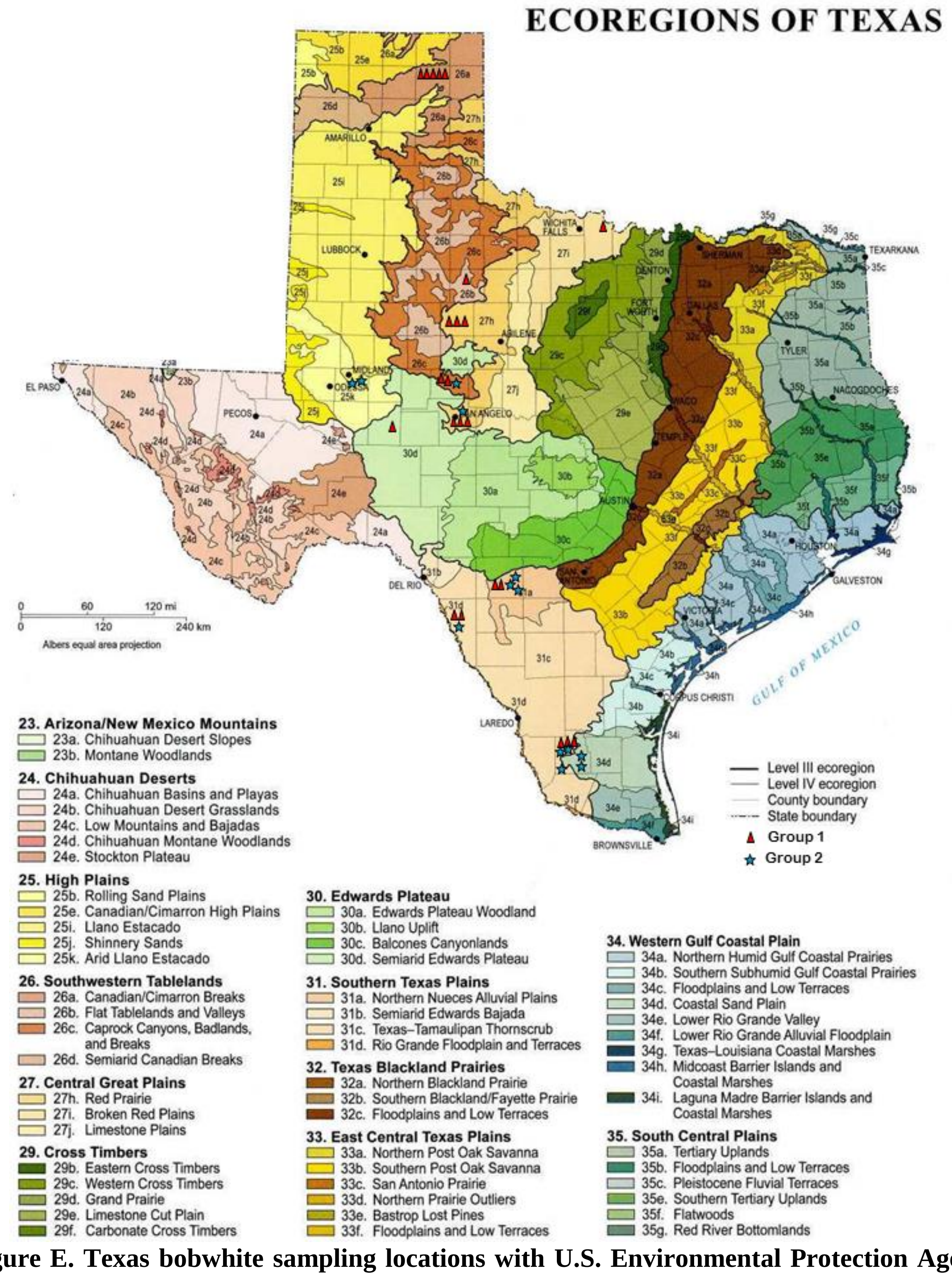

Figure E. Texas bobwhite sampling locations with U.S. Environmental Protection Agency level III ecoregions (adapted from http://archive.epa.gov/wed/ecoregions/web/html/level_iii_iv-2.html). Red triangles represent the collection sites for Group 1 bobwhites. Blue stars represent the collection sites for Group 2 bobwhites.

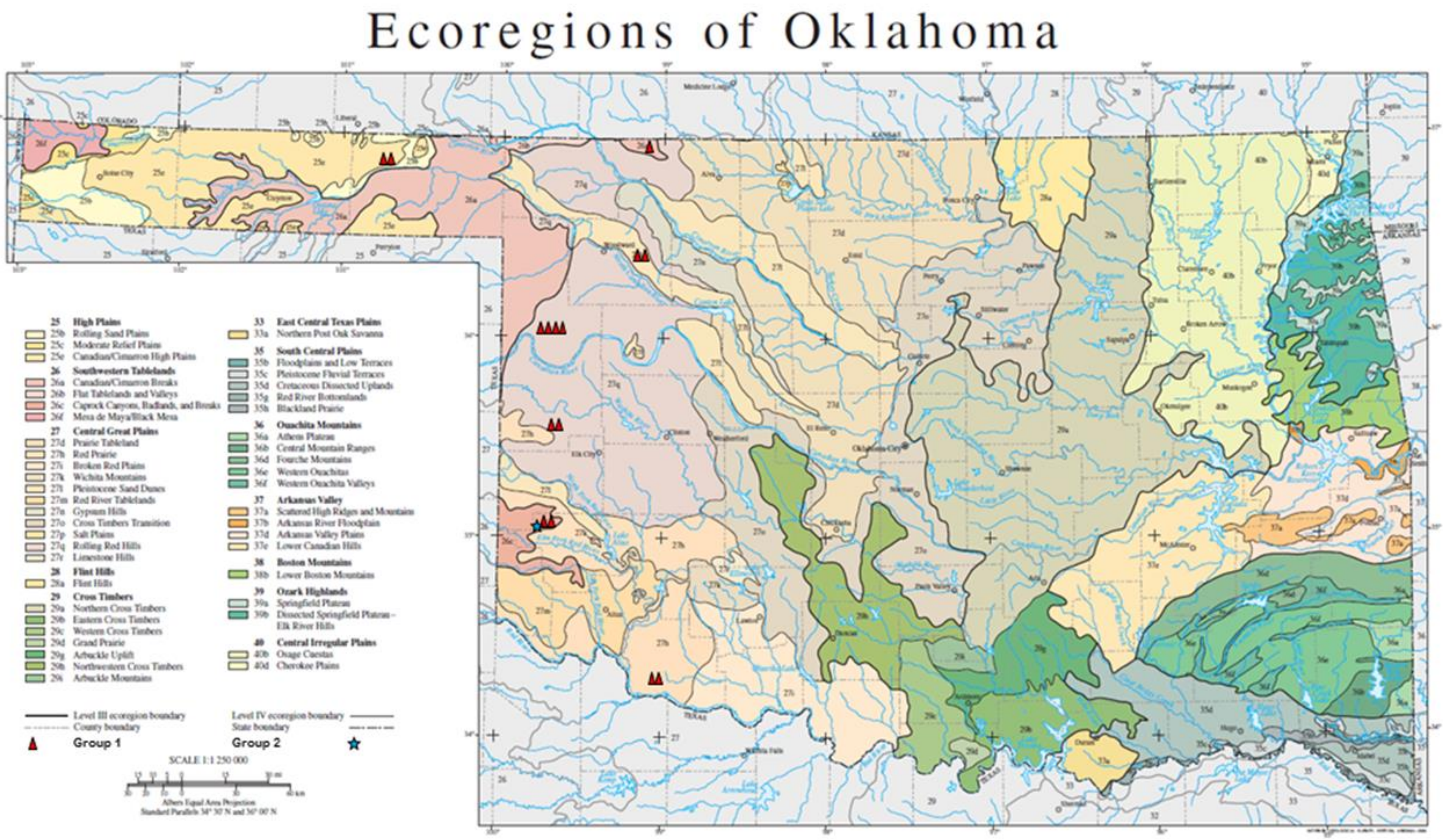

Figure F. Oklahoma bobwhite sampling locations with U.S. Environmental Protection Agency level III ecoregions (adapted from http://archive.epa.gov/wed/ecoregions/web/html/level_iii_iv-2.html). Red triangles represent the collection sites for Group 1 bobwhites. Blue stars represent the collection sites for Group 2 bobwhites.
